# A qualitative exploration of quality assurance for standardized patient in China

**DOI:** 10.3389/fmed.2026.1851094

**Published:** 2026-07-09

**Authors:** Lai Kun Tong, Yue Yi Li, Mio Leng Au, Choi Peng Wong, Wai I. Ng

**Affiliations:** Kiang Wu Nursing College of Macau, Macau, Macao SAR, China

**Keywords:** China, qualitative research, quality assurance, simulation-based education, standardized patient

## Abstract

**Background:**

The educational value of standardized patient (SP) programs depends on the quality of SP performance, which in turn requires appropriate quality assurance (QA) mechanisms. However, empirical descriptions of how QA systems are organized and maintained in the Chinese context remain limited.

**Objective:**

To qualitatively examine structures, processes, challenges, and improvement strategies related to QA for SP programs in China from multiple stakeholder perspectives.

**Methods:**

An exploratory qualitative study was conducted using three focus groups and 16 individual interviews (*N* = 23) with SP program coordinators/educators, clinical educators, and experienced SP from 12 institutions across four economic regions. Data were analyzed using thematic analysis.

**Results:**

Five themes described key QA-related practices and experiences. (1) Upstream quality control in recruitment and admission, including targeted recruitment, multi-dimensional entry criteria, diverse recruitment channels, and scale adjustment. (2) Standardized yet differentiated training, comprising modular curricula, flexible training formats, educator team development, standardized materials, and tailored strategies by SP type. (3) Closed-loop assessment and monitoring through post-training evaluation, ongoing supervision, periodic re-training, and graded management. (4) Key challenges, including performance inconsistency, workforce instability, resource constraints, and cross-cultural, ethical, and technological issues. (5) Optimization strategies, including enhanced standardization, incentive and management systems, resource integration, scenario-specific measures, and iterative evaluation.

**Conclusion:**

The findings describe a context-responsive QA practices and operational experiences characterized by structured processes and continuous refinement within prevailing resource conditions. These insights may support the development and maintenance of SP programs in similar settings.

## 1 Introduction

A standardized patient (SP) is an individual who has undergone specialized training to consistently and authentically simulate clinical presentations (1). SP-based simulation offers a controlled and risk-free environment (2, 3) that facilitates the development of clinical skills, communication proficiency, and diagnostic reasoning without compromising patient safety (4, 5). The educational effectiveness of this approach is dependent on the quality of SP performance (6–8), highlighting the necessity for a comprehensive quality assurance (QA) framework. Such a framework encompasses systematic processes, including recruitment and training, to ensure SP reliably achieves intended learning outcomes (9–11).

Although best practices for QA in SP programs have been well established in developed regions, research on how such systems are conceptualized, implemented, and sustained within the Chinese context remains scarce. Existing studies primarily focus on the feasibility and effectiveness of using SP in specific teaching or assessment scenarios (12), but rarely examine the underlying structures and processes that ensure quality. Given that China’s medical education ecosystem differs substantially from that of developed countries (13), there is an urgent need for an in-depth, qualitative exploration of the experiences, perspectives, and challenges encountered by key stakeholders, in order to inform QA practices tailored to the Chinese context.

This qualitative study is aimed to address the existing knowledge gap by examining current practices in QA for SP in China, identifying challenges encountered, and analyzing strategies employed to overcome these barriers. The findings will inform the development of a context-specific, evidence-based QA practices designed to the sustainable and high-quality advancement of SP-based education in China.

## 2 Methods

### 2.1 Study design

An exploratory qualitative design was employed, incorporating both group and individual interviews. The choice of an exploratory approach was informed by the lack of prior research on QA for SP programs in China.

### 2.2 Study setting and participants

Purposive sampling was adopted to select three institutions for group interviews, each representing distinct paradigms of SP programs in China. The first was the first institution to launch SP training in China and nationally recognized for mature SP integration into medical/nursing education, and residency training. The second was a government-led regional simulation center, initiated and funded by the local Health Commission, which operates a centralized SP program providing standardized recruitment, training, and resource sharing for affiliated hospitals and universities across the region. The third is one of the major training institutions in China for competition and foreign language SP. For individual interviews, participants were recruited from eight institutions stratified by China’s four economic regions (Eastern, Central, Western, Northeastern), with two institutions selected per region, to ensure representation of varying resource capacities, geographic contexts, and SP program maturity levels. Notably, institutional representatives and SPs were initially intended to be recruited from the same institutions. However, following one institutional representative’s interview, they indicated that their institution was on holiday and recommended a nearby institution with a comparable SP program for SP recruitment. This adjustment resulted in a final total of nine unique institutions contributing participants to individual interviews.

A total of 23 participants were enrolled, covering three core stakeholder groups to capture multi-dimensional perspectives on SP QA. Three group interviews (2–3 participants per group) were conducted, one at each key institution, with each group comprising 1 SP program coordinators/educators (responsible for designing, implementing, and monitoring SP recruitment, training, and QA processes) and 1–2 clinical educators/assessors (who regularly use SP in undergraduate education, postgraduate training, or licensing examinations). Individual interviews included 16 participants: 8 institutional stakeholders (SP program coordinators, educators, and clinical educators from the 8 stratified institutions, with 1 participant per institution and ≥5 years of direct involvement in SP program management) and 8 experienced SP (≥2 years of service) were recruited. Eligibility criteria for all participants included direct experience with SP programs (recruitment, training, use, or service as an SP), the ability to articulate detailed insights on QA practices, willingness to participate in audio-recorded interviews, and proficiency in Mandarin or Cantonese.

### 2.3 Data collection

Data collection was conducted by three trained researchers with complementary expertise to ensure rigor. The focus group moderator was a senior researcher with ≥15 years of clinical practice and medical education experience, plus 5 years of focus group facilitation experience in healthcare education research, responsible for guiding discussions, probing for depth, and managing group dynamics. The individual interviewer was a doctoral-level researcher with ≥15 years of medical education experience and 10 years of qualitative research expertise, tasked with administering individual interviews and adapting probes to participant roles. A third researcher with expertise in behavioral observation was present at all focus groups and individual interviews to document non-verbal cues (e.g., facial expressions, body language, tone of voice) that complemented audio-recorded data.

Focus group interviews were conducted online in August–December 2025, lasting 120–150 min per group. The moderator encouraged open dialogue to ensure all participants contributed, while the note-taker documented non-verbal interactions. Individual semi-structured interviews were conducted between August–December 2025, either in-person or via secure online platforms, lasting 30–80 min each. A semi-structured discussion guide, developed based on relevant literature, expert opinion and study objectives. The final interview guide is provided in Supplementary material 1. When the research team initially judged that data saturation had been reached, two additional institutional representatives and two SP were invited for further interviews. As no new information emerged from these interviews, data collection was concluded. The transcripts from these additional two institutional representatives and two SP were excluded from the analysis.

### 2.4 Data analysis

All interviews were audio-recorded and transcribed verbatim. Two undergraduate nursing students were responsible for verifying the accuracy of the transcripts. Data analysis was conducted using the Chinese transcripts, and all verbatim quotations presented in the manuscript were translated into English by bilingual experts. Analysis was conducted using NVivo 12 for data management. Data were analyzed using thematic analysis following Braun and Clarke’s six-step framework (14). (1) Familiarization: The first author (with more than 5 years of simulation experience) repeated reading of transcripts, notes, and document summaries, and recorded initial thoughts and preliminary observations during reading. (2) Coding: The first author segmented the data into meaningful units and assigned corresponding codes. For example, the excerpt “We recruit new SPs primarily through referrals from existing members of our pool.” was initially coded as “Recruit via internal referrals.” A total of 114 initial codes were generated (Supplementary material 2). These codes were not treated as fixed building blocks but as provisional interpretive devices subject to revision. (3) Generating themes: Codes were sorted into 22 candidate themes. For instance, codes related to “Public recruitment via official websites,” “Public recruitment via campus electronic bulletin boards,” “Joint recruitment with universities,” “Joint recruitment with hospital departments,” “Recruit social volunteers via community outreach,” “Organize volunteer recruitment campaigns” were clustered to form the initial concept of Multi-layer Recruitment Channels. (4) Reviewing themes: Each candidate theme was reviewed against coded extracts to ensure internal consistency; four candidate themes were collapsed or split. For instance, “Standardized SP Training” and “Differentiated SP Training” were combined into “Standardized & Differentiated SP Training.” (5) Defining and naming themes: Final themes were defined by identifying their essence and scope conditions. For example, Theme 1 (“Upstream Quality Control in Recruitment and Admission”) was defined as: “Institutional efforts to filter SP quality before formal training, encompassing targeted recruitment, multidimensional screening, channel diversification, and dynamic scaling.” (6) Writing the report: Themes were narrated with embedded quotations, ensuring each theme was grounded in participants’ voices while analytically interpreted.

To guarantee research rigor, triangulation was adopted across different data sources and participant groups. Member checking was also carried out with 11 participants (3 focus group participants and 8 individual interviewees), who reviewed the extracted themes and interpretations to verify the authenticity and accuracy of analytical outcomes.

## 3 Results

### 3.1 Participants

A total of 23 participants from 12 institutions were interviewed, including 15 institutional representatives (Table 1). All institutional representatives were female. Among the SP, gender distribution was balanced.

**Table 1:** Participant demographics.

Characteristic	*n*
Institutional representative	
Institution type	
Hospital	9
University	4
Government	2
Gender	
Male	0
Female	15
Education	
Master’s degree	5
Doctoral degree	10
Region	
Eastern	6
Central	2
Western	5
Northeastern	2
Standardized patient	
Education	
High school	6
Bachelor’s degree	2
Gender	
Male	4
Female	4

### 3.2 Findings

#### 3.2.1 Theme 1: upstream quality control in recruitment and admission

##### 3.2.1.1 Subtheme 1.1: diversified yet targeted recruitment

Based on their teaching objectives, available resources, and requirements for workforce stability, participating institutions adopted diversified yet purposed-driven recruitment strategies. Across sites, priority was consistently given to individuals without medical training to minimize the risk that professional knowledge would influence role portrayal.

*We rarely recruit SP with a medical background because they tend to inadvertently integrate clinical knowledge into their role portrayal, which may lead to deviations from the expected scenario.* (Individual interview participant, employed at a hospital in the eastern region).

Internal personnel and students functioned as supplementary sources. Hospitals primarily recruited residents enrolled in standardized training programs and administrative staff to support stable participation.

*All residents in standardized training programs and teaching group leaders are directly included in our SP pool… which ensures their availability.* (Individual interview participant, employed at a hospital in the northeastern region).

*I had wanted to be a SP since my school days, but my nursing background disqualified me because the school excluded students with medical training. The institution I work for now does not have that restriction and mainly recruits staff, so I can finally serve as a* SP. (Female SP with a bachelor’s degree).

Universities relied mainly on student SP, particularly those without medical training.

*All the SP we use are student SP without a medical background… Our goal at that time was explicit—to recruit students from our own university.* (Individual interview participant, employed at a hospital in the western region).

Socially stable groups-such as retirees and civil servants-were also recruited as long-term reserves due to flexible schedules and perceived reliability.

*Most of our young SP are either freelancers or employees from stable institutions such as civil service departments.* (Focus group participant, employed at the first institution in China to launch SP training).

##### 3.2.1.2 Subtheme 1.2: multi-dimensional entry criteria

All institutions established four dimensions admission criteria—basic competencies, professional attitude, physical condition, and background screening—to ensure the baseline level of quality.

For basic competencies (language proficiency, memory, and communication), SP involved in history-taking scenarios were required to demonstrate clear articulation and adaptive conversational skills. By contrast, SP assigned to physical-examination scenarios were prioritized according to overall physical fitness.

*For SP in history-taking sessions, we pay more attention to their occupational backgrounds, such as experience as teachers or salespersons… We highly value verbal expression and communication skills*…*For SP participating in physical-examination sessions, we place greater emphasis on their general physical conditions, preferring those with as few chronic diseases as possible.* (Focus group participant, employed at the first institution in China to launch SP training).

A strong professional attitude-particularly enthusiasm and a sense of responsibility-was widely viewed as an essential, and intrinsic motivation was emphasized as a selection principle.

*We hope to recruit as many dedicated volunteers as possible.* (Focus group participant, employed at an institution that trains SP for competitions and foreign language education).

*We would not want individuals who take on this role only for monetary compensation; first and foremost, they should believe that working as an SP contributes to the development of medical education*. (Individual interview participant, employed at a hospital in the central region).

Physical condition and background checks were treated as non-negotiable safety assurance. Annual health examinations were mandated, focusing on screening for infectious diseases.

*We arrange an annual physical examination for all SP, focusing primarily on infectious diseases. Moreover, our SP team is aging, with an average age over 55 and some in their 70s; we are concerned about potential health incidents during participation*. (Individual interview participant, employed at a university in the central northeastern).

Additional restrictions were implemented in specific contexts. For instance, one institution limited recruitment to internal candidates due to the demographic characteristics of its student population.

*Given the specific characteristics of our students, we must ensure their safety. Because we have many Tibetan students, recruitment is conducted internally, as external recruitment introduces too many uncertainties*. (Individual interview participant, employed at a hospital in the western region).

##### 3.2.1.3 Subtheme 1.3: multi-layer recruitment channels

To balance coverage and efficiency, institutions employed three primary pathways—internal referral, public recruitment, and collaborative partnerships. Internal referral (from staff, students, and current SP) was widely regarded as reliable and associated with lower attrition.

*We approached not only administrative staff but also shop owners near our campus. We later found this method did not provide satisfactory stability or time commitment; consequently, we have long-term engaged with administrative staff to work as* SP. (Individual interview participant, employed at a university in the central region).

*We recruit new SPs primarily through referrals from existing members of our pool. This approach extends beyond mere convenience; it leverages guanxi. When a trusted SP endorses a new recruit, I can be confident they understand our unwritten norms.* (Individual interview participant, employed at a university in the eastern region).

Public recruitment broadened outreach and increased candidate diversity through official websites, WeChat public accounts, and campus bulletin.

*Recruitment advertisements for SP are posted on our WeChat official accounts and internal information boards.* (Focus group participant, employed at the first institution in China to launch SP training).

*I saw the recruitment information for standardized patients on the school’s electronic bulletin board.* (Male SP with a bachelor’s degree).

Collaborative partnership leveraged resource integration and promoted long-term stability through cooperation with universities, hospitals, communities, and volunteer organizations.

*Recruitment is organized as volunteer drives: student volunteers are recruited regularly through university student associations; volunteers are co-recruited with hospital nursing departments; and social volunteers are recruited through ongoing community outreach, universities for the elderly, and WeChat official accounts.* (Focus group participant, employed at an institution that trains SP for competitions and foreign language education).

##### 3.2.1.4 Subtheme 1.4: dynamic scale adjustment

In response to teaching demands, assessment schedules, and workforce stability, institutions dynamically adjusted their recruitment scale to prevent both resource redundancy and shortages. For routine operational, a core pool was maintained to ensure readiness for emergent or high-volume tasks.

*We have trained a relatively stable team of volunteer SP, including more than 150 volunteers competent in both Chinese and English.* (Focus group participant, employed at an institution that trains SP for competitions and foreign language education).

For short-term or project-specific needs, targeted recruitment was used to support large-scale examinations or specialized activities.

*For Objective Structured Clinical Examination (OSCE), we organize dedicated open recruitment.* (Individual interview participant, employed at a university in the eastern region).

#### 3.2.2 Theme 2: standardized and differentiated training

##### 3.2.2.1 Subtheme 2.1: modular and tiered training content

Training was organized into four modules-basic knowledge, role-specific skills, case simulation, and emergency response-with tiered adjustments by SP types (history-taking, physical-examination, or communication). The basic knowledge module clarified SP role positioning, essential medical concepts, and scenario-specific protocols.

*The priority during SP training is to clarify their role positioning.* (Individual interview participant, employed at a hospital in the eastern region).

*At that time, none of us had a medical background. The training covered basic medical knowledge, including anatomy and physiology fundamentals, medical terminology, and typical clinical symptoms.* (Male SP with a high school diploma).

The role-specific skills module targeted competencies in performance, assessment, and feedback, reflecting SP’ three primary functions.

*We evaluate their performance from the perspectives of performer, assessor, and feedback provider, conduct on-site demonstrations based on real clinical cases, and organize group discussions to facilitate skill enhancement.* (Individual interview participant, employed at a hospital in the northeastern region).

The case-simulation module emphasized repeated practice and instructor guidance to improve proficiency and ensure performance consistency across encounters.

*Proficiency is mainly achieved through continuous guidance; crucially, SP must maintain relatively stable and consistent performance across all interactions with different students, and this is the top priority.* (Individual interview participant, employed at a university in the central region).

The emergency-response module prepared SP to manage unexpected or off-script situations. Training focused on appropriate responses to questions beyond the scope of the case.

*We also provide training for abnormal scenarios, such as when students raise questions completely irrelevant to the given case. and we teach SP how to respond appropriately in such circumstances.* (Individual interview participant, employed at a hospital in the eastern region).

##### 3.2.2.2 Subtheme 2.2: flexible duration and format

Training duration and delivery were adapted to SP types (community SP, student SP, in-house staff SP) and purpose (teaching, assessment, project), to balance feasibility and effectiveness. Community SP often required long-term, structured preparation.

*Our training consists of at least 60 class hours; they attend training twice a week, two evenings each time.* (Focus group participant, employed at the first institution in China to launch SP training).

Student SP received shorter, intensive sessions.

*(Student SP training) each session is relatively short-about one class hour. Later, student familiarize themselves with cases, interpret, and memorizing them, and practice repeatedly, usually 2 h per day… Students were generally able to memorize two cases per day.* (Individual interview participant, employed at a hospital in the western region)

For in-house staff SP, training was streamlined due to institutional familiarity.

*Since these SP are our faculty members, we provide them with the scripts and rehearse cases with them while we act as examinees. If they can perform without major issues, we approve them.* (Individual interview participant, employed at a university in the central region).

Training formats were similarly diverse, integrating online and offline learning, as well as centralized and group-based approaches. Online modules supported the delivery of foundational knowledge, particularly for SP unable to attend on-site sessions.

*Many SP really cannot come to the on-site sessions, so some courses can be completed online.* (Individual interview participant, employed at a hospital in the central region).

Core skills were predominantly taught through in-person, hands-on practice.

*Pre-assessment training… Since we mainly use SP for assessments, and the content of each assessment is completely different, we provide SP with special targeted pre-assessment training for each case.* (Focus group participant, employed at a government-led regional simulation center).

Case-based practice was conducted in small groups to enhance standardization and role consistency.

*During the case training, we group* SP. *For example, those around 60 years old may be assigned to portray diseases presenting with cough, such as COPD. We train with the same case within a group to ensure adequate mastery.* (Focus group participant, employed at the first institution in China to launch SP training).

##### 3.2.2.3 Subtheme 2.3: professional educator team building

The professional competence of SP educators was considered critical. Institutions developed trainer teams via systematic selection, ongoing capacity-building, and experience transfer. Educator selection prioritized individuals with clinical experience and teaching expertise.

*In fact, we always implement an admission mechanism for educators… they are generally clinical physicians and nurses who take on teaching responsibilities… They receive preliminary training, observe our SP training process, and then take up their posts.* (Focus group participant, employed at the first institution in China to launch SP training).

To support ongoing educator development, institutions adopted multiple strategies to ensure educators’ knowledge and skills remained current. Ongoing development included external training to update approaches and case design, and internal seminars for shared problem-solving.

*We send teachers to learn the latest case design and training methods.* (Individual interview participant, employed at a hospital in the central region).

*We hold internal discussions to share skills for handling common SP-related issues*. (Individual interview participant, employed at a university in the central northeastern).

##### 3.2.2.4 Subtheme 2.4: standardized training materials

Standardized materials were viewed as essential for efficiency, reproducibility, and traceability. Scripts and rating scales were routinely standardized. When examiner-provided descriptions were overly technical, educators revised cases into SP-friendly scripts.

*The cases provided by examiners are often incomprehensible to* SP. *Our educators need to rewrite them into scripts that SP can understand.* (Focus group participant, employed at a government-led regional simulation center).

Each case was accompanied by a concise, user-friendly rating scale to support consistency in performance.

*Every case is equipped with its own rating scale.* (Focus group participant, employed at the first institution in China to launch SP training).

Institutions also emphasized the use of simulation props to enhance scenario authenticity. These included special-effects makeup, medical models, and a variety of scene-specific materials.

*We need to apply makeup to* SP. *For example, for a blast injury case, we turned a girl in her twenties into a grandmother in her seventies for a nursing skills competition scenario. In addition, we also prepare laboratory test reports, medicine boxes, and other items for our* SP. (Focus group participant, employed at a government-led regional simulation center).

##### 3.2.2.5 Subtheme 2.5: tailored strategies by SP type

Training priorities were differentiated by SP group. For community SP (generally without medical backgrounds), emphasis was placed on basic medical literacy and role immersion.

*If the participants have no medical background, we will provide them with basic medical knowledge learning, such as understanding typical clinical symptoms.* (Focus group participant, employed at an institution that trains SP for competitions and foreign language education).

*We guide SP to enter the patient’s role and consider what the patient would experience. Preoperative patients, for instance, are often anxious; we ask SP to incorporate likely questions arising from that anxiety.* (Individual interview participant, employed at a university in the western region).

For student SP, who are more prone to improvisation, the focus was on discipline and performance consistency, with simplified content to avoid unnecessary clinical elaboration.

*(For nurse students acting as SP) we reiterate the rules repeatedly before the exam…Focus on memorizing the core information of cases instead of excessive medical knowledge training*. (Individual interview participant, employed at a university in the eastern region)

For in-house staff SP, who may incorporate clinical identities into performances, training emphasized role-switching and neutrality.

*(For in-house staff acting as SP) there are also issues that need attention, (we emphasize to SP) that they should remain neutral.* (Individual interview participant, employed at a university in the central region)

#### 3.2.3 Theme 3: SP: closed-loop assessment and monitoring

##### 3.2.3.1 Subtheme 3.1: post-training assessment

Institutions implemented a training–assessment–admission mechanism, following a principle of broad entry but strict progression. Assessments covered three dimensions—performance, scoring accuracy, and feedback ability—conducted in simulated station environments.

*We simulate a station-based scenario, where SP perform their roles and score the candidates while assessment instructors also score at the station. We then examine any significant discrepancy between SP and instructor scores. We also evaluate SP’ performance, communication, and memory, as well as their competence in evaluation and feedback.* (Individual interview participant, employed at a hospital in the central region).

SP meeting standards were admitted to the long-term pool; those who did not pass were offered one opportunity to retake the assessment.

*After completing our training and passing the assessment, these SP will be included in our long-term SP pool.* (Individual interview participant, employed at a hospital in the western region).

*If we do not pass, we only have one opportunity to retake it.* (Female SP with a high school diploma).

##### 3.2.3.2 Subtheme 3.2: ongoing process monitoring

During service, institutions employed on-site supervision, video review, and multi-stakeholder feedback to support real-time correction and maintain quality. Educators monitored performance and intervened when necessary. All encounters were recorded for *post-hoc* review, enabling timely communication with SP and examiners.

*During the exam… if an SP’s performance is unsatisfactory, reminders will be given to the SP through the backstage, behind the one-way mirror.* (Individual interview participant, employed at a university in the eastern region).

*We record everything. As an educator, I can also retrieve the videos to observe the performance of our* SP. *If any problems arise, we will communicate with the SP and examiners in a timely manner.* (Focus group participant, employed at a government-led regional simulation center).

Multi-stakeholder feedback included input from students, examiners, SP educators, and SP themselves, which was integrated into regular year reviews and forums to discuss issues and identify solutions.

*We routinely collect student feedback on SP and invite clinical instructors/examiners to rate their performance… Each academic year we hold a forum of instructors, SP educators, student representatives and all SP to review the data, identify problems and agree on improvements.* (Individual interview participant, employed at a university in the western region).

##### 3.2.3.3 Subtheme 3.3: periodic re-training

Regular retraining (annual or quarterly) was implemented to prevent skill decline, update knowledge, and ensure alignment with evolving teaching needs.

*We run an annual refresher that targets the specific skills SP underperformed in the previous year, building on the original training.* (Focus group participant, employed at a government-led regional simulation center).

Targeted sessions were held prior to major examinations for new or case-specific requirements, including case explanation, evaluation, and on-site simulation practice.

*(Before formal examinations) As SP we had to attend at least a half-day of training on any new case, covering case briefings, assessment criteria, and hands-on simulation practice.* (Female SP with a high school diploma)

##### 3.2.3.4 Subtheme 3.4: quality grading and incentives

A quality grading system enabled dynamic adjustment of SP classifications and served as a motivational mechanism.

*We classify SP into different grades; there is a grading system… If any SP consistently fails to receive positive feedback from our examiners, we may eliminate them from the pool or provide them with additional training.* (Focus group participant, employed at a government-led regional simulation center).

#### 3.2.4 Theme 4: core challenges

##### 3.2.4.1 Subtheme 4.1: poor consistency

Consistency across performance, scoring, and feedback was a central challenge. Participants noted substantial individual differences in skills and case comprehension, as well as intra-individual fluctuations across time and scenarios.

*SP perform differently in the morning and afternoon…mood may* var*y, so individual consistency is an issue.* (Focus group participant, employed at a government-led regional simulation center).

##### 3.2.4.2 Subtheme 4.2: high turnover and last-minute drop-outs

Workforce stability was suboptimal, with temporary absenteeism and long-term attrition disrupting teaching and assessment. Some SP permanently withdrew due to personal or professional changes; others requested leave on examination days due to unexpected events, necessitating last-minute replacements.

*The turnover rate is quite high… Sometimes they become mothers, change jobs, or switch the nature of their work, and thus no longer have time to serve as* SP. (Focus group participant, employed at an institution that trains SP for competitions and foreign language education).

*Over the years, we have encountered many incidents during exams, such as SP having to take emergency leave due to sudden illness or being accidentally injured by candidates, which necessitates last-minute replacements.* (Focus group participant, employed at a government-led regional simulation center).

##### 3.2.4.3 Subtheme 4.3: resource constraints

Resource shortages in funding, faculty, and equipment constrained institutional efforts to refine the SP quality assurance system. First, limited funding restricted the recruitment and incentivization of community SP. Many institutions reported tight budgets, resulting in low labor fees and minimal benefits.

*Currently, there is no such thing as allowances and benefits. We may occasionally provide some labor fees, but we cannot offer them to our in-house staff. We can probably give labor fees to students, but there is no way to provide them to our own employees.* (Individual interview participant, employed at a hospital in the eastern region).

Second, insufficient faculty resources undermined the depth and coverage of SP training. The lack of full-time educators meant that existing faculty had to assume additional responsibilities, often without dedicated departmental support.

*There is no dedicated department for SP; the training of SP is undertaken by our teachers who teach diagnostics.* (Individual interview participant, employed at a university in the central region).

Third, insufficient equipment further diminished scenario authenticity. Participants noted that the lack of advanced simulation devices and appropriate props constrained the fidelity of SP performances.

*If we need to simulate procedures such as puncture or subcutaneous injection, we cannot do it on our SP, which is also a limitation.* (Focus group participant, employed at a government-led regional simulation center).

##### 3.2.4.4 Subtheme 4.4: cross-cultural, ethical, and technological risks

In special scenarios such as cross-cultural teaching, ethical protection, and technological integration, additional challenges emerged in ensuring the quality of SP. First, cross-cultural teaching was hindered by linguistic and cultural barriers. Many community SP lacked adequate English proficiency and cultural familiarity required to support international learners, leading to concerns regarding communication effectiveness and learner acceptance.

*The number of community SP with sufficient English proficiency remains limited for international students… Most of our international students are from India, and we are concerned about issues of acceptance.* (Focus group participant, employed at the first institution in China to launch SP training).

Second, ethical concerns were prominent due to insufficient safeguards for SP privacy and psychological wellbeing. Participants described risks such as privacy breaches and the cumulative psychological burden associated with repeatedly portraying emotionally or physically distressing conditions.

*An SP may portray a hypertensive or anxious patient throughout the day, which is fatiguing. During physical examinations, SP must undress, so we need to protect their privacy.* (Focus group participant, employed at a government-led regional simulation center).

Third, integrating SP with advanced simulation technologies presented considerable challenges. Coordination between SP performance and physiological data displayed by high-fidelity simulators was inconsistent, undermining the authenticity and coherence of hybrid simulation scenarios.

*We previously attempted to use SP together with high-fidelity simulators, but their performance failed to align with the data displayed by the equipment; for example, fluctuations in blood pressure and oxygen saturation were not matched by SP responses.* (Individual interview participant, employed at a university in the eastern region).

#### 3.2.5 Theme 5: optimization strategies

##### 3.2.5.1 Subtheme 5.1: enhanced standardization

Consistency in performance, scoring, and feedback was strengthened through unified standards and operational manuals. Some institutions reported publishing academic materials to clarify procedural requirements.

*We have already published a paper in an academic journal that includes an introduction to this course.* (Focus group participant, employed at an institution that trains SP for competitions and foreign language education).

Assigning fixed roles or case matches to individual SP further enhanced stability by increasing familiarity and reducing variability across cycles.

*We assign at least one fixed case to each* SP. *For example, if an SP acts as a patient with a cough, they will consistently play this role across several teaching cycles.* (Focus group participant, employed at the first institution in China to launch SP training).

##### 3.2.5.2 Subtheme 5.2: better incentives and management

Attrition and last-minute absences were addressed via material and non-material incentives and structured backup mechanisms. Material incentives included increased labor compensation and additional benefits. In parallel, a systematic backup SP reserve was developed to mitigate last-minute absences.

*All SP participating in our training are awarded academic credits, and those involved in assessments receive financial compensation… Affiliated hospitals of universities can also provide free physical examinations for* SP. (Individual interview participant, employed at a hospital in the western region).

*For our large-scale examinations, we deploy backup SP at a minimum ratio of 10:1.* (Focus group participant, employed at a government-led regional simulation center).

##### 3.2.5.3 Subtheme 5.3: internal and external resource integration

Resource constraints were mitigated through inter-institutional collaboration, including a regional SP resource-sharing platform that centralized management and allocation, reduced duplication, and enabled city-wide reservation and use.

*To avoid duplicated effort and cut costs, we created a city-wide shared pool of SP, simulation facilitators and examiners that any local hospital or university can book on demand.* (Focus group participant, employed at a government-led regional simulation center).

##### 3.2.5.4 Subtheme 5.4: special-scene solutions

Targeted countermeasures were developed to address risks encountered in special scenarios and to ensure the quality of SP performance. For cross-cultural teaching, institutions implemented language and cultural adaptation training and recruited multilingual SP.

*(To recruiting non-Chinese SP) We mainly recruit international students from our university, and we also collaborate with a partner university to recruit some of their international students.* (Focus group participant, employed at an institution that trains SP for competitions and foreign language education)

To safeguard ethical considerations, mechanisms for privacy protection and psychological support were established. Enhancements included improved privacy protocols and access to counseling resources.

*If an SP is fatigued, we will replace them with a backup SP; after the assessment, we will communicate with them and provide desensitization therapy to help them relax.* (Focus group participant, employed at a government-led regional simulation center).

*During physical examinations, we were given shorts and tank tops to ensure privacy.* (Male SP with a high school diploma).

For technological integration, institutions delineated the complementary roles of SP and simulation equipment to maintain coherence in hybrid simulation scenarios.

*When SP are used with high-fidelity simulators… students complete technical operations on the simulators, while SP are responsible for situational communication and humanistic care.* (Individual interview participant, employed at a university in the eastern region).

##### 3.2.5.5 Subtheme 5.5: dynamic evaluation and continuous improvement

Through multi-source evaluation and closed-loop refinement, the quality of SP performance was continuously enhanced. A four-way dynamic appraisal system—incorporating evaluations from students, examiners, SP educators, and SP self-ratings—was established.

*Certificate holders enter our volunteer bank and undergo ongoing evaluation by learners, faculty, and the training team.* (Focus group participant, employed at an institution that trains SP for competitions and foreign language education).

Identified weaknesses triggered targeted improvement initiatives. For example, when consistency briefings alone proved insufficient, institutions introduced mandatory role-immersion rehearsals in which SP educators posed multiple probing questions to assess the depth of SP case embodiment.

*When consistency briefings proved insufficient, we added mandatory role-immersion rehearsals in which educators fire multiple questions to verify deep case embodiment.* (Individual interview participant, employed at a hospital in the eastern region).

## 4 Discussion

This study explored the quality assurance system of SP in medical and nursing education through a qualitative approach, identifying five core themes: upstream quality control in recruitment and admission, standardized and differentiated training, closed-loop assessment and monitoring, core challenges, and optimization strategies. The findings not only enrich the empirical evidence of SP management but also provide practical implications for refining SP quality assurance mechanisms in clinical teaching.

### 4.1 Institutional dependence and Guanxi networks: the cultural logic behind the “internalization” strategy in SP recruitment

The study found that institutions showed a strong preference for recruiting “insiders” (e.g., standardized training residents, administrative staff, enrolled students) and relying on “relationship-based referrals,” while viewing social stability (e.g., retirees, civil servants) as another important criterion. This internalized and relationship-oriented recruitment strategy stands in clear contrast to Western SP management models, which typically emphasize open public recruitment (15). This divergence reflects two structural characteristics of the institutional environment in which the study is situated.

First, the strategy stems from an institutional pursuit of control and reliability. Within China’s resource-constrained and high-stakes medical education system (13), ensuring the stability and availability of the SP workforce is essential for program sustainability. Relying on internal personnel and existing networks significantly reduces the high turnover rate and coordination costs associated with external, unfamiliar SP (16), representing a rational, risk-reducing choice under limited resources.

Second, this strategy is deeply embedded in cultural norms that emphasize guanxi (relationships) and trust. Through referrals from acquaintances or internal conversion, the relationship between recruiters and SP extends beyond a simple labor contract (17): additional layers of organizational affiliation and interpersonal reciprocity create implicit obligations and trust (18, 19), which in turn strengthen SP’ sense of responsibility and engagement.

However, this strategy also carries the risk of homogeneity, potentially reducing diversity in SP demographics such as age, occupation, and socioeconomic background (20). This may limit the authenticity of scenarios that require representation of diverse social groups (21). Overall, the findings reveal that SP management is not a value-neutral “technical process”; rather, the initial stage of recruitment is already shaped by local institutional logic and cultural preferences.

### 4.2 The dialectical integration of standardization and differentiation: contextualized pedagogical intelligence in SP training

This study delineates a fundamental paradox in SP training: on the one hand, institutions strive for maximal consistency through modularized content, standardized training materials, and certification-based assessments. On the other hand, they simultaneously adjust training duration, content, and emphasis flexibly according to SP type—community SP, student SP, and in-house staff SP. This strategy of “standardized framework with differentiated implementation” represents a locally grounded solution to the inherent tension between fidelity and feasibility widely discussed in SP education theory (16, 22).

For community SP with no medical background, training prioritizes basic biomedical literacy and role immersion (3). For student SP—who are more prone to improvisation and deviation from scripts—discipline, adherence to standardized performance, and reduction of unnecessary clinical elaboration become central requirements (23, 24). For in-house staff SP, whose professional identities may interfere with lay-patient performance, emphasis is placed on role-switching skills and maintaining neutrality. This approach reflects educators’ sophisticated contextualized pedagogical intelligence, which goes beyond merely applying international training manuals. Instead, it relies on careful diagnosis of diverse learners’ cognitive characteristics, motivational structures, and potential sources of performance bias to inform targeted pedagogical intervention (24, 25).

Such practices align with theories of adaptive expertise, which propose that experts adjust strategies flexibly in response to varying contextual demands (26). These findings also indicate heightened expectations for the professional development of SP educators: they are not simply implementers of standardized procedures, but designers capable of group-level diagnosis and differentiated instructional design.

### 4.3 Closed-loop management and continuous optimization: a systems-engineering approach under resource constraints

This study presents a comprehensive closed-loop practices encompassing recruitment, training, assessment, monitoring, retraining, and graded incentives, reflecting a clear systems-engineering mindset and an orientation toward continuous improvement. Particularly in contexts where resources are limited, the establishment of refined procedures, dynamic adjustments, and multisource feedback mechanisms represents an innovative effort to maximize the efficiency of existing resources. For example, the development of a city-level SP resource-sharing platform provides an innovative solution to the fragmentation inherent in the traditional “danwei (work unit) -system,” shifting practice from institution-level “isolated efforts” to regional “collaborative governance” (27, 28).

However, the findings also reveal internal tensions within the system. Despite the establishment of elaborate quality-monitoring mechanisms—such as on-site supervision, video review, and four-party evaluation—SP performance inconsistency remains a persistent and challenging issue (29). This suggests that heavy reliance on technical and managerial controls alone may be insufficient to address the inherent variability of human performance (30, 31). Future research should explore how psychological and emotional support interventions can be integrated into the management cycle, thereby facilitating a shift from merely “controlling consistency” to actively “empowering stability” (32).

### 4.4 Emerging challenges and ethical frontiers: localized responses in the context of globalization and technological integration

The challenges identified in cross-cultural teaching, ethical protection, and technological integration position the discussion of SP management at the forefront of global medical education and technology ethics. Strategies such as recruiting bilingual SP and providing cultural adaptation training represent essential localized responses as Chinese medical education advances toward greater internationalization. Concerns regarding SP privacy protection (e.g., during physical examinations), psychological fatigue, and the potential long-term emotional impact of portraying traumatic scenarios closely mirror the growing global emphasis on ethical standards and occupational wellbeing within the SP field (9, 33). These findings suggest that SP programs in China have moved beyond the earlier pursuit of “establishment” and “standardization,” and are transitioning toward deeper attention to “quality” and “humanistic considerations.”

With regard to risks associated with technological integration—such as inconsistencies between SP performance and data generated by high-fidelity simulators—institutions have adopted strategies that clearly delineate the complementary roles of human SP and simulation technologies. By assigning technical procedures to simulators while positioning SP to deliver communication and humanistic care (34), programs have developed a clear and practical framework to address the coordination challenges inherent in hybrid simulation scenarios.

## 5 Limitations

This study has several limitations. First, the purposive sampling of institutions and participants—although intended to capture heterogeneity—constrains the generalizability of the findings beyond similar program contexts. Second, the perspectives of certain key stakeholders (e.g., learners who interact with SPs) were not included, which may have limited triangulation on issues of performance quality and learner outcomes. Third, all data derived from interviews are subject to recall and social-desirability bias, potentially leading participants to portray practices in a more favorable light.

## 6 Conclusion

This study examined key management practices across the recruitment, training, assessment, and continuous improvement processes of SP programs, and described an SP QA system shaped by the specific context of medical and nursing education in China. The findings indicate that effective SP management requires coordinated system design, iterative quality monitoring, and a balance between standardization and flexibility, rather than reliance on isolated measures. These insights offer a practical reference for SP program development in resource-limited environments and contribute to broader discussions on organizational learning and contextual adaptation within simulation-based education. Future research using longitudinal or cross-cultural comparative designs may further evaluate the applicability and effectiveness of these system components across diverse settings.

## Data Availability

The raw data supporting the conclusions of this article will be made available by the authors, without undue reservation.
