# Supplementary material for: A qualitative exploration of quality assurance for standardized patient in China
Source: Front Med (Lausanne). 2026 Jul 9;13:1851094. doi: 10.3389/fmed.2026.1851094 (PMC13391949; doi:10.3389/fmed.2026.1851094)
Supplement: Supplementary file 1 [file Data_Sheet_1.docx]

Supplementary Material

**Supplementary 1: Interview Guide**

**For Institutional Representatives**

1. Do you incorporate standardized patients in your teaching?
2. Could you describe how standardized patients are used in your instructional activities?
3. How do you recruit standardized patients?
4. What requirements or qualifications do you expect from standardized patients?
5. Do you provide training for standardized patients? If so, could you describe the training content?
6. Who is responsible for conducting the SP training? How is the training implemented in practice? What is the duration and format of the training? Is any form of assessment required? Do you have a dedicated curriculum for SP training?
7. After completing the training, are standardized patients generally able to meet the teaching requirements? Could you provide examples?
8. What aspect of the SP training process has left the deepest impression on you?
9. What is the most memorable experience you have had while using standardized patients in teaching?
10. Is there anything else you would like to add regarding the topic of standardized patients?

**For Standardized Patients**

1. Have you ever served as a standardized patient?
2. What motivated you to take on the role of a standardized patient?
3. Could you describe your experience portraying a standardized patient?
4. Did you receive any training before participating as a standardized patient?
   - *If yes:* What types of training did you receive? Do you consider the training sufficient? What additional training, if any, would you recommend?
5. Did you encounter any challenges while portraying a standardized patient? Please provide examples.
6. Is there anything else you would like to add regarding your experience as a standardized patient?

# Supplementary 2: Coding Matrix: From raw data to codes, sub-themes and themes

| **Raw Data Excerpt** | **Initial Codes** | Sub-themes | Themes |
| --- | --- | --- | --- |
| We rarely recruit SP with a medical background because they tend to inadvertently integrate clinical knowledge into their role portrayal, which may lead to deviations from the expected scenario. | Prefer candidates without medical background | Diversified yet Targeted Recruitment | Upstream Quality Control in Recruitment and Admission |
| We rarely recruit SP with a medical background because they tend to inadvertently integrate clinical knowledge into their role portrayal, which may lead to deviations from the expected scenario. | Medical knowledge distorts role portrayal |  |  |
| All residents in standardized training programs and teaching group leaders are directly included in our SP pool… which ensures their availability. | Recruit standardized training residents |  |  |
| I had wanted to be a SP since my school days, but my nursing background disqualified me because the school excluded students with medical training. The institution I work for now doesn’t have that restriction and mainly recruits staff, so I can finally serve as a SP. | Recruit institutional administrative staff |  |  |
| All the SP we use are student SP without a medical background… Our goal at that time was explicit—to recruit students from our own university. | Recruit university students as SP |  |  |
| I had wanted to be a SP since my school days, but my nursing background disqualified me because the school excluded students with medical training. | Exclude applicants with medical training |  |  |
| Socially stable groups-such as retirees and civil servants-were also recruited as long-term reserves due to flexible schedules and perceived reliability. | Recruit retirees for long-term reserve |  |  |
| Most of our young SP are either freelancers or employees from stable institutions such as civil service departments. | Recruit civil servants for stable participation |  |  |
| Socially stable groups-such as retirees and civil servants-were also recruited as long-term reserves due to flexible schedules and perceived reliability. | Prioritize socially stable groups for recruitment |  |  |
| For SP in history‑taking sessions, we pay more attention to their occupational backgrounds… We highly value verbal expression and communication skills. | Require strong verbal and communication skills for history-taking SP | Multi-dimensional Entry Criteria |  |
| For SP participating in physical-examination sessions, we place greater emphasis on their general physical conditions, preferring those with as few chronic diseases as possible. | Prioritize good physical fitness for physical-examination SP |  |  |
| We hope to recruit as many dedicated volunteers as possible. | Value enthusiasm and sense of responsibility |  |  |
| We would not want individuals who take on this role only for monetary compensation; first and foremost, they should believe that working as an SP contributes to the development of medical education. | Prioritize intrinsic motivation over financial reward |  |  |
| We arrange an annual physical examination for all SP, focusing primarily on infectious diseases. | Require annual physical examination for all SP |  |  |
| We arrange an annual physical examination for all SP, focusing primarily on infectious diseases. | Screen for infectious diseases in health checks |  |  |
| Given the specific characteristics of our students, we must ensure their safety. Because we have many Tibetan students, recruitment is conducted internally, as external recruitment introduces too many uncertainties. | Adopt internal recruitment for special student groups |  |  |
| All institutions established four dimensions admission criteria—basic competencies, professional attitude, physical condition, and background screening—to ensure the baseline level of quality. | Conduct strict background screening for safety |  |  |
| We recruit new SPs primarily through referrals from existing members of our pool. | Recruit via internal referrals | Multi-layer Recruitment Channels |  |
| Internal referral (from staff, students, and current SP) was widely regarded as reliable and associated with lower attrition. | Internal referral features high reliability and low attrition |  |  |
| This approach extends beyond mere convenience; it leverages guanxi. When a trusted SP endorses a new recruit, I can be confident they understand our unwritten norms. | Rely on interpersonal connections in referrals |  |  |
| To balance coverage and efficiency, institutions employed three primary pathways—internal referral, public recruitment, and collaborative partnerships through official websites, WeChat public accounts, and campus bulletin. | Public recruitment via official websites |  |  |
| Recruitment advertisements for SP are posted on our WeChat official accounts and internal information boards. | Public recruitment via WeChat official accounts |  |  |
| I saw the recruitment information for standardized patients on the school’s electronic bulletin board. | Public recruitment via campus electronic bulletin boards |  |  |
| student volunteers are recruited regularly through university student associations. | Joint recruitment with universities |  |  |
| volunteers are co-recruited with hospital nursing departments. | Joint recruitment with hospital departments |  |  |
| social volunteers are recruited through ongoing community outreach. | Recruit social volunteers via community outreach |  |  |
| Recruitment is organized as volunteer drives. | Organize volunteer recruitment campaigns |  |  |
| social volunteers are recruited through ongoing community outreach, universities for the elderly, and WeChat official accounts. | Recruit volunteers from senior universities |  |  |
| We have trained a relatively stable team of volunteer SP, including more than 150 volunteers competent in both Chinese and English. | Maintain a stable core SP team | Dynamic Scale Adjustment |  |
| In response to teaching demands, assessment schedules, and workforce stability, institutions dynamically adjusted their recruitment scale to prevent both resource redundancy and shortages. | Adjust recruitment scale according to work demands |  |  |
| For Objective Structured Clinical Examination (OSCE), we organize dedicated open recruitment. | Conduct targeted recruitment for examinations |  |  |
| In response to teaching demands, assessment schedules, and workforce stability, institutions dynamically adjusted their recruitment scale to prevent both resource redundancy and shortages. | Avoid manpower surplus and shortage |  |  |
| The priority during SP training is to clarify their role positioning. At that time, none of us had a medical background. The training covered basic medical knowledge, including anatomy and physiology fundamentals, medical terminology, and typical clinical symptoms. | Training on role positioning and basic medical knowledge | Modular and Tiered Training Content | Standardized and Differentiated Training |
| We evaluate their performance from the perspectives of performer, assessor, and feedback provider, conduct on-site demonstrations based on real clinical cases, and organize group discussions to facilitate skill enhancement. | Training on performance, assessment and feedback skills |  |  |
| Proficiency is mainly achieved through continuous guidance; crucially, SP must maintain relatively stable and consistent performance across all interactions with different students, and this is the top priority. | Repeated case simulation practice |  |  |
| Proficiency is mainly achieved through continuous guidance; crucially, SP must maintain relatively stable and consistent performance across all interactions with different students, and this is the top priority. | Require consistent performance in role-playing |  |  |
| We also provide training for abnormal scenarios, such as when students raise questions completely irrelevant to the given case. | Training on handling unexpected scenarios |  |  |
| We also provide training for abnormal scenarios, such as when students raise questions completely irrelevant to the given case... and we teach SP how to respond appropriately in such circumstances. | Train SP to answer off-script questions |  |  |
| Our training consists of at least 60 class hours; they attend training twice a week, two evenings each time. | Long-cycle training for community SP | Flexible Duration and Format |  |
| (Student SP training) each session is relatively short-about one class hour. Later, student familiarize themselves with cases, interpret, and memorizing them, and practice repeatedly, usually two hours per day… Students were generally able to memorize two cases per day. | Short intensive training for student SP |  |  |
| Since these SP are our faculty members, we provide them with the scripts and rehearse cases with them while we act as examinees. If they can perform without major issues, we approve them. | Simplified training for institutional staff SP |  |  |
| Many SP really cannot come to the on-site sessions, so some courses can be completed online. | Online learning for basic knowledge |  |  |
| Pre-assessment training… Since we mainly use SP for assessments, and the content of each assessment is completely different, we provide SP with special targeted pre-assessment training for each case. | Offline practical training for core skills |  |  |
| During the case training, we group SP. For example, those around 60 years old may be assigned to portray diseases presenting with cough, such as COPD. We train with the same case within a group to ensure adequate mastery. | Group-based case training |  |  |
| Pre-assessment training… Since we mainly use SP for assessments, and the content of each assessment is completely different, we provide SP with special targeted pre-assessment training for each case. | Case-specific training before assessments |  |  |
| In fact, we always implement an admission mechanism for educators… they are generally clinical physicians and nurses who take on teaching responsibilities. | Select trainers with clinical and teaching experience | Professional Educator Team Building |  |
| They receive preliminary training, observe our SP training process, and then take up their posts. | Pre-job training and observation for new trainers |  |  |
| We send teachers to learn the latest case design and training methods. | External training for updated teaching methods and case design |  |  |
| We hold internal discussions to share skills for handling common SP-related issues. | Internal seminars for experience and problem sharing |  |  |
| The cases provided by examiners are often incomprehensible to SP. Our educators need to rewrite them into scripts that SP can understand. | Revise professional cases into easy-to-understand scripts | Standardized Training Materials |  |
| Every case is equipped with its own rating scale. | Equip each case with standardized rating scales |  |  |
| We need to apply makeup to SP. For example, for a blast injury case, we turned a girl in her twenties into a grandmother in her seventies for a nursing skills competition scenario. | Use special makeup for scenario simulation |  |  |
| In addition, we also prepare laboratory test reports, medicine boxes, and other items for our SP. | Prepare medical props and inspection documents |  |  |
| If the participants have no medical background, we will provide them with basic medical knowledge learning, such as understanding typical clinical symptoms. | Teach basic medical knowledge to community SP | Tailored Strategies by SP Type |  |
| We guide SP to enter the patient’s role and consider what the patient would experience. Preoperative patients, for instance, are often anxious; we ask SP to incorporate likely questions arising from that anxiety. | Train community SP for role immersion |  |  |
| (For nurse students acting as SP) we reiterate the rules repeatedly before the exam. | Strengthen rule compliance for student SP |  |  |
| Focus on memorizing the core information of cases instead of excessive medical knowledge training. | Simplify case content for student SP |  |  |
| (For in-house staff acting as SP) there are also issues that need attention. | Train staff SP to switch professional roles |  |  |
| (we emphasize to SP) that they should remain neutral. | Require role neutrality for staff SP |  |  |
| Institutions implemented a training–assessment–admission mechanism, following a principle of broad entry but strict progression. | Implement training-assessment-admission mechanism | Post-training Assessment | SP: Closed-loop Assessment and Monitoring |
| We simulate a station-based scenario, where SP perform their roles and score the candidates while assessment instructors also score at the station. We then examine any significant discrepancy between SP and instructor scores. We also evaluate SP’ performance, communication, and memory, as well as their competence in evaluation and feedback. | Assess performance, scoring and feedback ability |  |  |
| We simulate a station-based scenario, where SP perform their roles and score the candidates while assessment instructors also score at the station. We then examine any significant discrepancy between SP and instructor scores. | Compare scores between SP and instructors |  |  |
| After completing our training and passing the assessment, these SP will be included in our long-term SP pool. | Admit qualified SP into official talent pool |  |  |
| If we don’t pass, we only have one opportunity to retake it. | Offer one retest opportunity for unqualified SP |  |  |
| During the exam… if an SP’s performance is unsatisfactory, reminders will be given to the SP through the backstage, behind the one-way mirror. | On-site supervision and real-time reminder | Ongoing Process Monitoring |  |
| We record everything. As an educator, I can also retrieve the videos to observe the performance of our SP. | Video recording and post-review |  |  |
| We routinely collect student feedback on SP and invite clinical instructors/examiners to rate their performance. | Collect feedback from multiple stakeholders |  |  |
| Each academic year we hold a forum of instructors, SP educators, student representatives and all SP to review the data, identify problems and agree on improvements. | Hold annual meetings to summarize and solve problems |  |  |
| We run an annual refresher that targets the specific skills SP underperformed in the previous year, building on the original training. | Organize regular annual refresher training | Periodic Re-training |  |
| We run an annual refresher that targets the specific skills SP underperformed in the previous year, building on the original training. | Targeted training for weak competencies |  |  |
| (Before formal examinations) As SP we had to attend at least a half-day of training on any new case. | Conduct new case training before examinations |  |  |
| (Before formal examinations) As SP we had to attend at least a half-day of training on any new case, covering case briefings, assessment criteria, and hands-on simulation practice. | Carry out case explanation and simulation before exams |  |  |
| We classify SP into different grades; there is a grading system. | Establish SP performance grading system | Quality grading and Incentives |  |
| A quality grading system enabled dynamic adjustment of SP classifications and served as a motivational mechanism. | Adjust SP classification dynamically |  |  |
| If any SP consistently fails to receive positive feedback from our examiners, we may eliminate them from the pool or provide them with additional training. | Arrange supplementary training or eliminate underperforming SP |  |  |
| Participants noted substantial individual differences in skills and case comprehension, as well as intra‑individual fluctuations across time and scenarios. | Inter-individual differences in role performance and case understanding | Poor Consistency | Core Challenges |
| SP perform differently in the morning and afternoon…mood may vary, so individual consistency is an issue. | Intra-individual performance fluctuations |  |  |
| Consistency across performance, scoring, and feedback was a central challenge. | Inconsistent scoring and feedback standards |  |  |
| The turnover rate is quite high. | High overall SP turnover rate | High Turnover and Last-minute Drop-outs |  |
| Sometimes they become mothers, change jobs, or switch the nature of their work, and thus no longer have time to serve as SP. | Resignation due to personal work and life changes |  |  |
| Over the years, we have encountered many incidents during exams, such as SP having to take emergency leave due to sudden illness. | Emergency absence on examination days |  |  |
| Over the years, we have encountered many incidents during exams, such as SP having to take emergency leave due to sudden illness or being accidentally injured by candidates, which necessitates last‑minute replacements. | Temporary absence caused by illness or injury |  |  |
| Over the years, we have encountered many incidents during exams… which necessitates last‑minute replacements. | Frequent need for temporary replacements |  |  |
| Currently, there is no such thing as allowances and benefits. We may occasionally provide some labor fees, but we cannot offer them to our in-house staff. | Insufficient funding and limited remuneration | Resource Constraints |  |
| There is no dedicated department for SP; the training of SP is undertaken by our teachers who teach diagnostics. | Lack of independent SP management department |  |  |
| There is no dedicated department for SP; the training of SP is undertaken by our teachers who teach diagnostics. | SP training undertaken by part-time teachers |  |  |
| Third, insufficient equipment further diminished scenario authenticity. | Shortage of simulation equipment and props |  |  |
| If we need to simulate procedures such as puncture or subcutaneous injection, we cannot do it on our SP, which is also a limitation. | Limitations in on-body clinical simulation |  |  |
| The number of community SP with sufficient English proficiency remains limited for international students. | Insufficient English ability among community SP | Cross-cultural, Ethical, and Technological Risks |  |
| Most of our international students are from India, and we are concerned about issues of acceptance. | Cultural adaptation issues with international students |  |  |
| During physical examinations, SP must undress, so we need to protect their privacy. | Privacy risks during physical examination simulation |  |  |
| An SP may portray a hypertensive or anxious patient throughout the day, which is fatiguing. | Psychological burden from long-term role-playing |  |  |
| We previously attempted to use SP together with high‑fidelity simulators, but their performance failed to align with the data displayed by the equipment. | Mismatch between SP performance and simulator data |  |  |
| Coordination between SP performance and physiological data displayed by high‑fidelity simulators was inconsistent, undermining the authenticity and coherence of hybrid simulation scenarios. | Poor coordination in hybrid simulation scenarios |  |  |
| We have already published a paper in an academic journal that includes an introduction to this course. | Compile unified documents and academic guidelines | Enhanced Standardization | Optimization Strategies |
| We assign at least one fixed case to each SP. For example, if an SP acts as a patient with a cough, they will consistently play this role across several teaching cycles. | Assign fixed roles and cases to individual SP |  |  |
| Assigning fixed roles or case matches to individual SP further enhanced stability by increasing familiarity and reducing variability across cycles. | Reduce performance differences and improve stability |  |  |
| All SP participating in our training are awarded academic credits, and those involved in assessments receive financial compensation… Affiliated hospitals of universities can also provide free physical examinations for SP. | Provide material rewards including labor fees and physical exams | Better Incentives and Management |  |
| All SP participating in our training are awarded academic credits. | Offer non-material incentives such as academic credits |  |  |
| a systematic backup SP reserve was developed to mitigate last‑minute absences. | Build a backup SP reserve team |  |  |
| For our large‑scale examinations, we deploy backup SP at a minimum ratio of 10:1. | Allocate backup personnel according to fixed ratio |  |  |
| To avoid duplicated effort and cut costs, we created a city-wide shared pool of SP, simulation facilitators and examiners that any local hospital or university can book on demand. | Establish regional SP resource sharing platform | Internal and External Resource Integration |  |
| To avoid duplicated effort and cut costs, we created a city-wide shared pool of SP, simulation facilitators and examiners that any local hospital or university can book on demand. | Realize cross-institutional resource co-management |  |  |
| To avoid duplicated effort and cut costs, we created a city-wide shared pool of SP, simulation facilitators and examiners that any local hospital or university can book on demand. | Reduce repeated construction and operating costs |  |  |
| (To recruiting non‑Chinese SP) We mainly recruit international students from our university, and we also collaborate with a partner university to recruit some of their international students. | Recruit multilingual SP for cross-cultural teaching | Special-scene Solutions |  |
| institutions implemented language and cultural adaptation training and recruited multilingual SP for cross‑cultural teaching. | Conduct language and cultural adaptation training |  |  |
| During physical examinations, we were given shorts and tank tops to ensure privacy. | Improve privacy protection measures |  |  |
| If an SP is fatigued, we will replace them with a backup SP; after the assessment, we will communicate with them and provide desensitization therapy to help them relax. | Provide psychological support and relaxation guidance |  |  |
| When SP are used with high‑fidelity simulators… students complete technical operations on the simulators, while SP are responsible for situational communication and humanistic care. | Clarify respective duties of SP and simulation devices |  |  |
| institutions delineated the complementary roles of SP and simulation equipment to maintain coherence in hybrid simulation scenarios. | Optimize hybrid simulation scenario design |  |  |
| Certificate holders enter our volunteer bank and undergo ongoing evaluation by learners, faculty, and the training team. | Implement evaluation system | Dynamic Evaluation and Continuous Improvement |  |
| Identified weaknesses triggered targeted improvement initiatives. | Carry out targeted improvements for identified problems |  |  |
| When consistency briefings proved insufficient, we introduced mandatory role‑immersion rehearsals in which educators fire multiple questions to verify deep case embodiment. | Add mandatory role immersion rehearsals |  |  |
| When consistency briefings proved insufficient, we introduced mandatory role‑immersion rehearsals in which educators fire multiple probing questions to assess the depth of SP case embodiment. | Assess case mastery through on-site questioning |  |  |
